# ERK Dephosphorylation through MKP1 Deacetylation by SIRT1 Attenuates RAS-Driven Tumorigenesis

**DOI:** 10.3390/cancers12040909

**Published:** 2020-04-08

**Authors:** Ok-Seon Kwon, Haeseung Lee, Yun-Jeong Kim, Hyuk-Jin Cha, Na-Young Song, Mi-Ok Lee

**Affiliations:** 1 Stem Cell Convergence Research Center, Korea Research Institute of Bioscience and Biotechnology (KRIBB), Daejeon 34141, Korea; 2 Intellectual Information Team, Future Medicine Division, Korea Institute of Oriental Medicine, Daejeon 34054, Korea; 3 College of Pharmacy, Seoul National University, Seoul 08826, Korea; yunjkim36@gmail.com (Y.-J.K.); hjcha93@snu.ac.kr (H.-J.C.); 4 Research Institute of Pharmaceutical Sciences, Seoul National University, Seoul 08826, Korea; 5 Department of Oral Biology, Yonsei University College of Dentistry, Seoul 03722, Korea; 6 Department of Functional Genomics, KRIBB School of Bioscience, Korea University of Science and Technology (UST), Daejeon 34141, Korea

**Keywords:** SIRT1, RAS, ERK, MKP1, tumorigenesis, deacetylation

## Abstract

The role of Situin 1 (SIRT1) in tumorigenesis is still controversial due to its wide range of substrates, including both oncoproteins and tumor suppressors. A recent study has demonstrated that SIRT1 interferes in the Kirsten rat sarcoma viral oncogene homolog (KRAS)-driven activation of the Raf-mitogen-activated protein kinase (MAPK)/extracellular signal-regulated kinase (ERK) kinase (MEK)-ERK pathway, thereby inhibiting tumorigenesis. However, the molecular mechanism of SIRT1 as a tumor suppressor in RAS-driven tumorigenesis has been less clearly determined. This study presents evidence that the ectopic expression of SIRT1 attenuates RAS- or MEK-driven ERK activation and reduces cellular proliferation and transformation in vitro. The attenuation of ERK activation by SIRT1 results from prompt dephosphorylation of ERK, while MEK activity remains unchanged. We identified that MKP1, a dual specific phosphatase for MAPK, was deacetylated by SIRT1. Deacetylation of MKP1 by direct interaction with SIRT1 increased the binding affinity to ERK which in turn facilitated inactivation of ERK. Taken together, these results suggest that SIRT1 would act as a tumor suppressor by modulating RAS-driven ERK activity through MKP1 deacetylation.

## 1. Introduction

Sirtuin 1 (SIRT1), a NAD^+^-dependent class III histone deacetylase (HDAC), serves as an important deacetylase in diverse biological processes such as aging, stress response, metabolism, cell cycle, and so on [1,2,3]. In particular, SIRT1 plays a controversial role in carcinogenesis as either a suppressor or a driver [4,5]. Cumulative evidence shows that SIRT1 promotes tumorigenesis by inhibiting diverse tumor suppressors such as p53 [6,7] and forkhead class O transcription factors [8] through direct deacetylation. The relatively high expression of *SIRT1* in diverse cancers [9,10] and its cancer-prone effects endorse the role of SIRT1 as a tumor driver [4,11]. On the other hand, the roles of SIRT1 in the maintenance of genetic stability and the DNA repair process have been demonstrated in not only in vitro models [12,13,14] but also in vivo mouse models [15]. In addition, Sirt1^+/−^ and p53^+/−^ mice develop more tumors in multiple tissues, suggesting that SIRT1 acts as a tumor suppressor [15]. Recently, it has been reported that SIRT1 suppresses Kirsten rat sarcoma viral oncogene homolog (KRAS)-driven lung tumorigenesis by interfering in KRAS-induced transcriptional programs [16]. However, the molecular mechanism of the tumor-suppressive role of SIRT1 in RAS-driven tumorigenesis has yet to be fully elucidated.

The RAS-mitogen-activated protein kinase (MAPK) pathway is the essential cellular signaling for proliferation, differentiation, and survival in response to mitogen signals [17,18,19]. Notably, 20–30% of all human tumors result from the overexpression or oncogenic mutations of RAS to render this signal pathway constitutively active, which causes uncontrolled proliferation, neoplastic transformation, and angiogenesis [20]. RAS transmits signals to activate Raf, leading to activation of extracellular signal-regulated kinase (ERK) through MAPK/ERK kinase (MEK), eventually promoting cyclin D1 expression and constant Rb hyperphosphorylation, which are responsible for self-sufficiency in the growth signal, one of the hallmarks of cancer [21,22]. On the other hand, upon oncogenic activation, anti-growth signals induce senescence or apoptosis to protect cells from oncogenic transformation [23]. Thus, inhibition of anti-growth signals is also supposed to be one of the key hallmarks of cancer. Activation of p38 MAPK upon oncogenic RAS activation is required for oncogenic stress responses [24,25] and simultaneously contributes to repressing ERK activity through suppressing MEK activity [26,27]. 

MAPK phosphatase 1 (MKP1), encoded by dual specificity phosphatase 1 (DUSP1), is identified as a primary phosphatase for deactivating MAPKs [28,29]. Induction of MKP1 through immediate early gene responses by MEK activation inhibits the ERK activity as a negative feedback of the RAS -Raf-MEK-ERK pathway [30]. In addition, the ectopic expression of MKP1 inhibits RAS-induced DNA synthesis [31]. The role of MKP1 as a tumor suppressor has been further supported by the evidence that suppression of *MKP1* expression occurs in advanced cancers with higher histological grades [32,33,34]. On the contrary, MKP1 is a phosphatase for MAPKs, such as p38 and ERK, to modulate the stress responses or apoptosis that serves as one of the important tumor surveillance programs [35]. Thus, the pro-tumorigenic role of MKP1 is also well recognized [36,37], which is further supported by its increased expression in various cancers [38,39]. The dichotomous role of MKP1 in cancer has been attributed to the deactivation of various substrates, such as ERK, p38, and JNK, depending on the diverse cellular contexts [34]. Notably, the acetylation of MKP1 enhances its interaction with p38, thereby increasing its phosphatase activity to deactivate p38 and subsequently inhibit innate immune responses [40]. 

In the present study, we provide evidence that the ectopic expression of SIRT1 delays RAS-induced tumorigenesis by attenuating ERK phosphorylation. Importantly, we find that SIRT1 deacetylates MKP1 to increase direct interaction to ERK, which sequentially results in the suppression of cellular proliferation through ERK dephosphorylation. Taken together, we suggest that SIRT1 would protect from RAS-driven tumorigenesis through MKP1 deacetylation.

## 2. Results

### 2.1. Higher Expression of SIRT1 Correlates to Better Prognosis in Human Cancers

**Figure 1:**
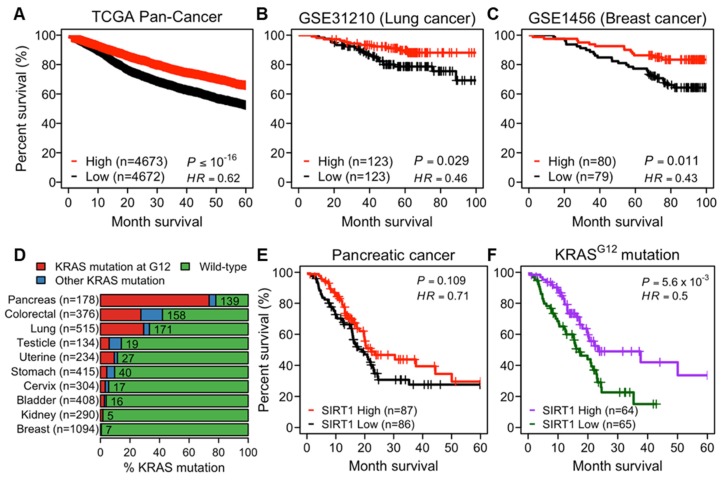
Protective role of *SIRT1* in human cancer. (**A**–**C**) The Kaplan–Meier curves showing overall survival (OS) of cancer patients stratified by *SIRT1*-high and -low groups. Patients were divided into *SIRT1*-high and -low expression groups, using the median *SIRT1* expression as a cutoff. OS stratified by *SIRT1*-high and -low groups in patients in The Cancer Genome Atlas (TCGA) pan-cancer cohort (**A**), lung (**B**) and breast (**C**) cancers in the Gene Expression Omnibus (GEO) datasets. (**D**) A bar graph showing the prevalence of *Kirsten rat sarcoma viral oncogene homolog (KRAS)* alterations across TCGA tumor samples grouped by tumor types. The information on *KRAS* alterations was obtained from cBioPortal. For visualization, ten cancer types including at least five patients with *KRAS* mutation at glycine 12 (G12) were shown. The number of patients with *KRAS* mutations is indicated on the bar graph. (**E**,**F**) The Kaplan–Meier curves showing OS stratified by *SIRT1*-high and -low groups in TCGA pancreatic cancer patients (**E**) and those carrying *KRAS G12* (**F**).

Considering the controversial role of SIRT1 in cancer, we evaluated the prognostic significance of SIRT1 by analyzing the correlation between *SIRT1* expression and overall survival (OS) of cancer patients. From The Cancer Genome Atlas (TCGA) tumor samples spanning 33 cancer types, we showed that the higher expression of *SIRT1* was strongly associated with favorable OS (Figure 1A and Figure S1A). This association was also observed in independent cohorts of lung and breast cancer patients (Figure 1B,C). Considering a previous report that increased *SIRT1* expression protects from KRAS-driven lung carcinogenesis [16], we further investigated RAS-dependency in the correlation between *SIRT1* expression and survival rates of patients with pancreatic, colorectal, and lung cancers, which frequently carry *KRAS* mutation(s), particularly on glycine 12 (Figure 1D). There were no significant differences in the survival rates between *SIRT1*-high and -low groups (Figure 1E and Figure S1B), or between *KRAS* mutant and wild-type groups (Figure S1C). However, a higher expression of *SIRT1* was associated with better survival probability in the *KRAS*-mutated patient group (Figure 1F and Figure S1D). Taken together, these survival analyses support the idea that higher *SIRT1* expression correlates to prolonged OS in patients with cancers, particularly those carrying *RAS* mutations.

### 2.2. SIRT1 Suppresses RAS-Driven Tumorigenic Activities In Vitro

**Figure 2:**
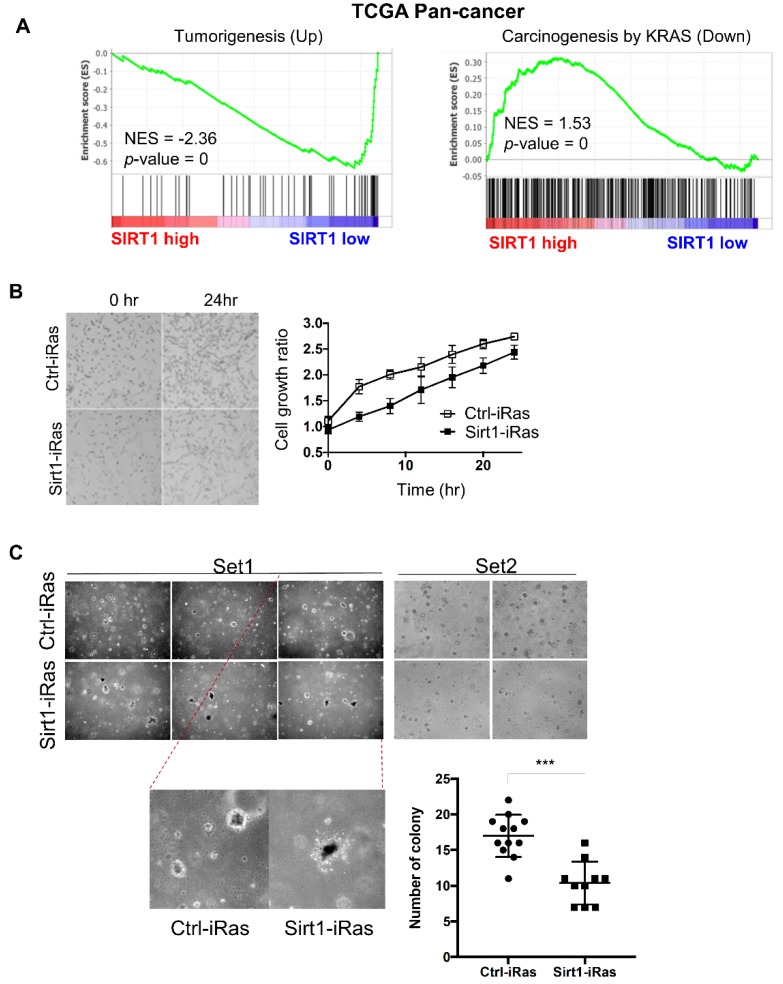
SIRT1 suppresses tumorigenesis. (**A**) Gene set enrichment analysis (GSEA) plots showing the enrichment of tumorigenesis-related genes in a ranked list of genes differentially expressed between *SIRT1*-high and -low groups in TCGA pan-cancer cohort (*n* = 9435). Tumorigenesis-related gene sets were obtained from MSigDB: ‘CROMER TUMORIGENESIS UP’ (left) and ‘IWANAGA CARCINOGENESIS BY KRAS PTEN DN’ (right). The normalized enrichment score (NES) and nominal p-value were calculated by the GSEA tool. (**B**) Comparison of growth rates between Ctrl-iRas and Sirt1-iRas cells. The cell proliferation rate was assessed by the JuLI stage at each time point. Representative images (left, original magnification, 40×) and growth curves (right) of Ctrl-iRas and Sirt1-iRas NIH3T3 cells. (**C**) The soft-agar assays were performed in Ctrl-iRas or Sirt1-iRas NIH3T3 cells with doxycycline (Dox) treatment. Representative images of soft-agar assay (left, original magnification, 40×) and a dot plot represents the number of colonies (right).

To further validate the correlation between *SIRT1* expression and tumorigenesis, we examined the transcript abundance of tumorigenic signatures in TCGA tumor patients. We divided 9345 patients into *SIRT1*-high and *SIRT1*-low groups based on the median expression of *SIRT1* (Figure S1A). Then, a gene set enrichment analysis (GSEA) was performed with tumorigenesis-related genes (Figure 2A). GSEA data revealed that a set of upregulated genes during tumorigenesis was markedly reduced in the *SIRT1*-high group, implying SIRT1 as a tumor suppressor (Figure 2A, left). On the contrary, a set of downregulated genes related to KRAS-driven tumorigenesis was significantly associated with higher expression of *SIRT1* (Figure 2A, right). In addition to RAS-dependency in the correlation between *SIRT1* expression and OS of cancer patients as shown in Figure 1F, this further suggests that SIRT1 can act as a more potent tumor suppressor particularly in RAS-driven tumorigenesis.

To evaluate an anti-tumorigenic effect of SIRT1 in cancers harboring *RAS* mutations, we established doxycycline (Dox)-inducible *HRAS^G12V^* oncogenic mutation cell lines by using a Tet-on system in control (Ctrl-iRas) or *SIRT1*-overexpressing (Sirt1-iRas) NIH3T3 cells, subsequently enriched by FACS sorting with fluorescence signals from humanized Kusabira-Orange protein (Figure S2A). Dox treatment effectively induced *HRAS* expression in both Ctrl-iRas and Sirt1-iRas cells, while *SIRT1* expression was significantly upregulated only in Sirt1-iRas cells (Figure S2B–E). However, *HRAS* expression itself was not able to alter *SIRT1* expression levels in Sirt1-iRas cells (Figure S2B,C,E). For subsequent experiments, we thus utilized these Ctrl-iRas and Sirt1-iRas cells to elucidate the role of SIRT1 in the HRAS-driven tumorigenic process.

As predicted, the cell proliferation rate was moderately reduced in Sirt1-iRas cells compared to Ctrl-iRas cells (Figure 2B). Consistently, the soft agar assay revealed that H-Ras-induced anchorage-independent growth was remarkably inhibited by SIRT1 upregulation (Figure 2C). All these results suggest that SIRT1 is capable of suppressing RAS-driven tumorigenic potential in NIH3T3 cells.

### 2.3. SIRT1 Inhibits RAS-MEK-ERK Axis via Dephosphorylation of ERK

**Figure 3:**
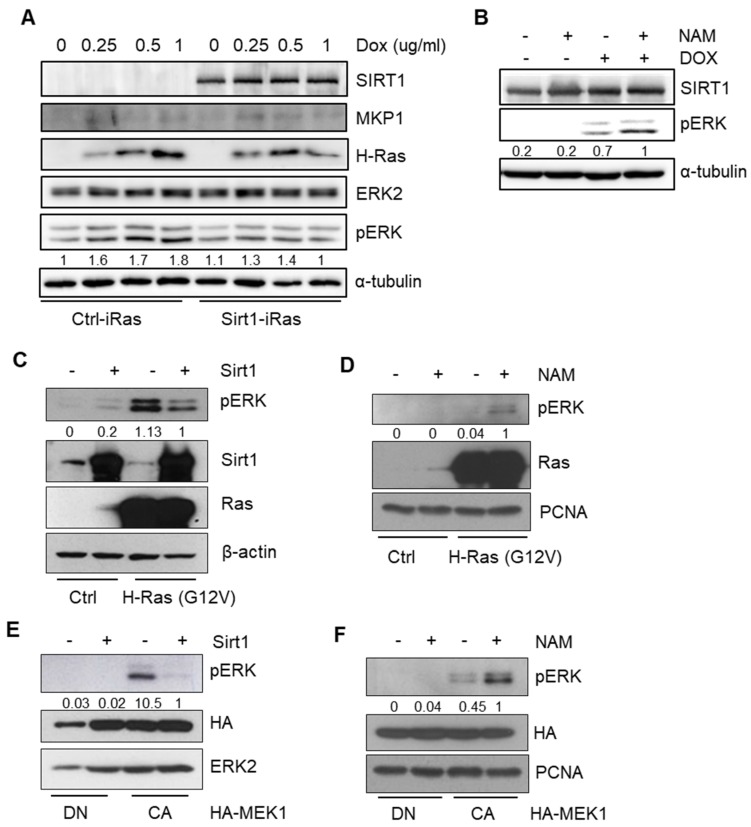
SIRT1 impairs RAS-mitogen-activated protein kinase (MAPK)/extracellular signal-regulated kinase (ERK) kinase (MEK)-ERK signaling. (**A**) Immunoblotting (IB) analysis shows phospho-ERK levels in Ctrl-iRas and Sirt1-iRas NIH3T3 cells. The cells were treated with Dox for 24 h at the indicated concentration. α-tubulin, a protein loading control for IB. (**B**) IB analysis of phospho-ERK in Sirt1-iRas NIH3T3 cells treated with or without nicotinamide (NAM) and Dox for 24 h. (**C**,**D**) IB reveals phospho-ERK levels after transfection with HRAS in the 293T cells depending on Sirt1 expression (**C**) or NAM treatment (**D**). β-actin and proliferating cell nuclear antigen (PCNA), protein loading controls for IB. (**E**,**F**) IB shows phospho-ERK levels after transfection with dominant negative (DN) or constitutively active (CA) MEK1 in the 293T cells depending on Sirt1 expression (**E**) or NAM treatment (**F**). ERK2 and PCNA, protein loading controls for IB. The whole western blot images please find in Figure S7.

We then investigated the molecular mechanisms underlying the inhibitory effects of SIRT1 on RAS-driven tumorigenesis. It has been recognized that constitutive activation of oncogenic RAS employs the MEK-ERK axis to amplify its pro-tumorigenic downstream signaling pathways [41]. In the present study, we also verified the HRAS-MEK-ERK axis in both Ctrl-iRas and Sirt1-iRas cell lines. Upon exposure to Dox, the phosphorylation kinetics of ERK were significantly impeded in Sirt1-iRas cells, unlike the gradual amplification that was found in Ctrl-iRas cells (Figure 3A), implying that SIRT1 can inhibit this RAS-MEK-ERK axis. In line with this notion, while a SIRT1 activator resveratrol (RSV) inhibited ERK phosphorylation in the E1A-Ras-MEF cells (Figure S3A), nicotinamide (NAM), a SIRT1 inhibitor, induced the phospho-ERK level to a remarkable extent in Sirt1-iRas and E1A-Ras-MEF cells (Figure 3B, Figure S3A). This NAM-induced dephosphorylation of ERK was also observed in human breast cancer MDA-MB-231 cells harboring *KRAS* mutation (Figure S3B). For further validation, the phosphorylation level of ERK was examined after the transient expression of constitutively active (CA) HRAS or MEK1. Constitutive activation of HRAS or MEK1 significantly augmented ERK phosphorylation, which was reduced by SIRT1 overexpression (Figure 3C,E). On the other hands, SIRT1 inhibition by NAM treatment successfully increased phospho-ERK expression in either HA-RAS or HA-MEK1 expressing cells (Figure 3D,F), implying that the deacetylase activity of SIRT1 can negatively regulate ERK phosphorylation in the RAS-MEK-ERK axis. Furthermore, the phosphorylation level of ERK was rapidly diminished in Sirt1-iRas cells compared to Ctrl-iRas after treatment with MEK1/2 inhibitor U0126 that blocks the MEK-ERK axis (Figure S3C). Thus, SIRT1 may enhance de-phosphorylation of ERK rather than modulate its phosphorylation. To sum up, these data suggest that SIRT1 suppresses the RAS-MEK-ERK axis through ERK dephosphorylation, exerting anti-tumorigenic activities. 

### 2.4. SIRT1 Dephosphorylates ERK through Deacetylation of MKP1

**Figure 4:**
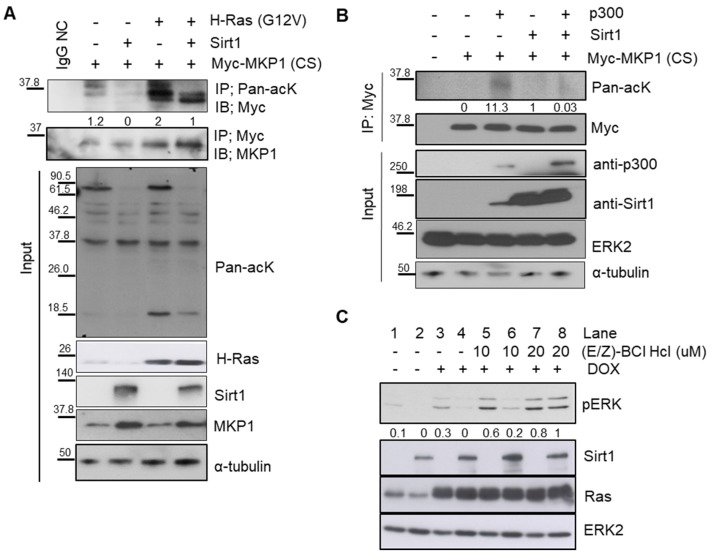
MAPK phosphatase 1 (MKP1) is responsible for SIRT1-dependent ERK dephosphorylation. (**A**) IB analysis of MKP1 expression and acetylation in 293T cells. Cells were transiently transfected with indicated vectors and then immunoprecipitation (IP) was performed with an anti-pan-acetyl lysine (acK) antibody, followed by IB with an anti-Myc antibody. IgG, a negative control for IP. (**B**) IB analysis of acetylation status of MKP1 upon overexpression of p300 and/or SIRT1 in 293T cells. IP was performed with an anti-Myc antibody, followed by IB with an anti-pan acK antibody. (**C**) IB analysis of phospho-ERK levels depending on SIRT1 expression in the presence or absence of a MKP1/6 inhibitor (E/Z)-BCl Hcl in Dox-exposed Ctrl-iRas (Lane# 1, 3, 5, and 7) and Sirt1-iRas (Lane# 2, 4, 6, and 8) cells. MKP1/6 inhibitor (E/Z)-BCl Hcl and Dox was treated for 3h. ERK2 and α-tubulin, protein loading controls for IB. The whole western blot images please find in Figure S7.

Next, how SIRT1 dephosphorylates ERK remained to be addressed. SIRT1 is a deacetylase, but not a phosphatase. It is well-known that phosphatase MKP1 directly catalyzes ERK dephosphorylation. We thus hypothesized that the phosphatase MKP1 could mediate SIRT1-dependent ERK dephosphorylation. Among 10 different MKPs, particularly in the case of MKP1, its phosphatase activity can be determined by its acetylation status [40,42]. In this regard, MKP1 was thought to be a plausible phosphatase responsible for SIRT1-dependent ERK dephosphorylation. To examine whether MKP1 is deacetylated by SIRT1, a pull-down assay was performed in 293T cells overexpressing SIRT1 as well as MKP1-CS, a catalytically inactive form of MKP1, to trap its substrates [43]. We found that SIRT1 directly bound to MKP1 (Figure S4A,B). Oncogenic HRAS promoted MKP1 acetylation, which was then attenuated by SIRT1 overexpression (Figure 4A). Moreover, SIRT1 reduced MKP1 acetylation (Figure 4B), while p300, a well-known acyl transferase, increased the MKP1 acetylation level (Figure S5). Based on these observations, it is evident that MKP1 is a novel target of SIRT1 deacetylase.

We further investigated whether this SIRT1-dependent deacetylation of MKP1 regulates ERK dephosphorylation, by utilizing (E/Z)-BCl Hcl, a small molecule that preferentially inhibits both MKP1 and MKP6. SIRT1 overexpression repressed HRAS-induced phosphorylation of ERK, but it was attenuated by (E/Z)-BCl Hcl treatment (Figure 4C). Taken together, these data support the idea that SIRT1 directly deacetylates MKP1, resulting in ERK dephosphorylation. 

### 2.5. SIRT1 Promotes Interaction between MKP1 and ERK via Its Deacetylase Activity

**Figure 5:**
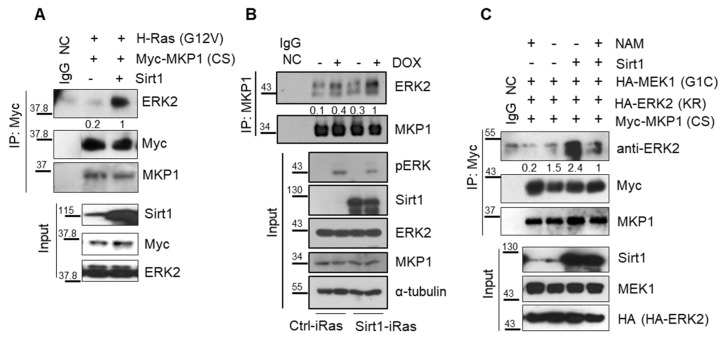
SIRT1 enhances interaction between MKP1 and ERK. (**A**) CA-RAS-overexpressing 293T cells were transfected with vectors containing Myc-tagged MKP1-CS plus or minus of SIRT1. IP was performed with an anti-Myc antibody, followed by IB with an anti-ERK2 antibody. IgG, a negative control for IP. (**B**) Co-IP was performed with an endogenous MKP1 in Ctrl-iRas and Sirt1-iRas cells treated with or without Dox for 3 h. (**C**) CA-MEK1-overexpressing 293T cells were transfected with Myc-tagged MKP1-CS plus or minus SIRT1, then treated with or without NAM. IP was performed with an anti-Myc antibody, followed by IB with an anti-ERK2 antibody. IgG NC, a negative control for IP. The whole western blot images please find in Figure S7.

We next explored how MKP1 deacetylation by SIRT1 modulates ERK phosphorylation status. Given a report that the acetylation site of MKP1 is lysine 57 residue in the kinase interacting motif of the conserved N-terminal domain, we hypothesized that SIRT1-dependent MKP1 deacetylation alters its binding affinity to ERK and subsequently affects ERK phosphorylation through its phosphatase activity [44]. To verify the interaction between MKP1 and ERK, we performed the immunoprecipitation (IP) analysis in 293T cells transfected with vectors containing CA-RAS and MKP1-CS in the presence or absence of SIRT1 ectopic expression. We found that SIRT1 increased the binding affinity between MKP1 and ERK under RAS overexpression (Figure 5A). Moreover, SIRT1 overexpression enhanced interaction between endogenous MKP1 and ERK in Dox-exposed Ctrl-iRas cells, resulting in diminished ERK phosphorylation (Figure 5B). This was further confirmed in a setting of constitutive activation of MEK1, a counterpart regulator of MKP1 phosphatase. SIRT1 promoted MKP1 binding to ERK, but this binding was impeded in the presence of NAM, a SIRT1 deacetylase inhibitor (Figure 5C). These results showed that SIRT1 intensifies the physical interaction between MKP1 and ERK, contributing to increased ERK dephosphorylation by MKP1. Herein, SIRT1-dependent MKP1 deacetylation might be crucial for the MKP1-ERK interaction.

**Figure 6:**
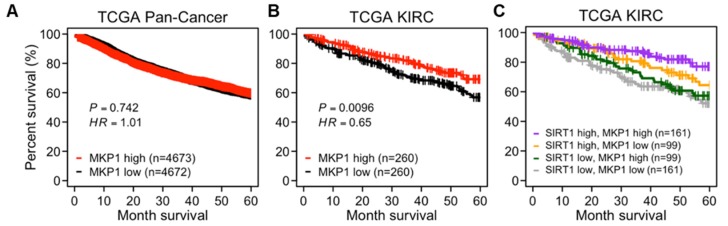
Prognostic significance of *MKP1* with *SIRT1* expression. (**A**–**C**) The Kaplan–Meier plots showing the OS of cancer patients in TCGA data. The OS stratified by *MKP1*-high and -low patient groups in a pan-cancer cohort (**A**); the OS stratified by *MKP1*-high and -low groups in patients of kidney renal clear cell carcinoma (TCGA KIRC) (**B**); the OS stratified by the combination of *SIRT1*-high/-low and *MKP1*-high/-low groups in patients of TCGA KIRC (**C**). HR and p-values derived from comparisons among individual groups are placed in Figure S6.

To evaluate the prognostic significance of MKP1, a survival analysis was performed using TCGA data. In a pan-cancer cohort, there was no difference in the OS based on the *MKP1* expression level (Figure 6A). However, in the case of kidney clear cell carcinoma (KIRC), where *MKP1* is most highly expressed compared to the other cancer types, the patient group with high *MKP1* expression exhibited better OS than those with low *MKP1* expression (Figure S6 and Figure 6B). Interestingly, the difference between these groups was more obvious when *SIRT1* was expressed abundantly (Figure 6C). These data support the idea that the combination of higher *MKP1* and higher *SIRT1* expression is strongly associated with a better prognosis. Although MKP1 itself is not a tumor suppressor, MKP1 might potentiate the anti-tumorigenic effect of SIRT1.

## 3. Discussion

Acetylation is one of the major post-translational modifications, including phosphorylation and methylation. So far, acetylation is most widely studied in the context of histone modification and the epigenetic regulations [45]. Recently, the non-histone protein acetylation is emerging as a cross-regulator in the epigenetic program as well as in protein functions [46]. Acetylation can be layered on the other protein modifications, which contributes to the complex and multiple control of protein functions. Thus, the acetylation-phosphorylation crosstalk is considered a new chemotherapeutic target that is particularly useful when either kinase or phosphatase is undruggable [47].

SIRT1, a NAD^+^-dependent class III histone deacetylase, can regulate tumorigenesis by modulating the acetylation-phosphorylation crosstalk. In this regard, it is well known that SIRT1 deacetylates non-histone proteins responsible for inflammation-driven carcinogenesis, such as STAT3 and p65 transcription factors. To switch on tumorigenic signals, phosphorylation is crucial for the activation of both transcription factors. Notably, their phosphorylation levels can be enhanced by acetylation status, consequently fortifying expression of pro-inflammatory and pro-tumorigenic genes [48,49]. However, SIRT1-dependent deacetylation reduces the phosphorylation levels of both STAT3 and p65, contributing to the fine-tuning of inflammation-driven tumorigenesis. 

Recently, SIRT1 has been demonstrated to suppress KRAS-driven lung carcinogenesis by inhibiting PI3K and MEK pathways [16]. According to the TCGA and Gene Expression Omnibus (GEO) databases, we also found that SIRT1 overexpression was correlated to better prognosis in human patients with cancers that frequently carry oncogenic *RAS* mutations (Figure 1 and Figure S1). Furthermore, our in vitro data supported the finding that SIRT1 overexpression markedly hampered HRAS-induced tumorigenic activities, indicating SIRT1 as a tumor suppressor in the oncogenic RAS activation setting as well. Upon RAS activation, MEK phosphorylates ERK to amplify oncogenic signal transduction, while MKP1 phosphatase dephosphorylates and deactivates ERK [17,20]. Interestingly, MKP1 phosphatase activity can be regulated by acetylation. In the present study, we first showed that MKP1 is a novel deacetylation target of SIRT1. Then, Sirt1-dependent MKP1 deacetylation enhanced the physical interaction between MKP1 and ERK, which led to the dephosphorylation of ERK in Ctrl-iRas cells. This suggests that MKP1 modulates the acetylation-phosphorylation crosstalk in the RAS-MEK-ERK cascade. However, there still remains a possibility that SIRT1 may recruit other target(s), other than MKP1, to regulate ERK phosphorylation, which thus requires further validation.

As mentioned above, it has been reported that the acetylation of MKP1 enhances p38 dephosphorylation, which seems to be the opposite of our findings about the acetylation-phosphorylation crosstalk in ERK regulation by MKP1. Rather, we supposed that acetylation status of MKP1 may decide its substrate preference. MKP1 has been reported to preferentially inactivate p38 and JNK MAPKs over ERK [50,51,52]. However, our data suggest that deacetylated MKP1 might readily interact with ERK, instead of p38 or JNK. Further investigation on the substrate specificity depending on MKP1 acetylation status is required to elucidate the role of MKP1 as a game-changer between ERK and p38 regulation.

## 4. Materials and Methods

### 4.1. Cell Culture and Establishment of Stable Cell Line

The 293T, MDA-MB-231, NIH3T3, and E1a/Ras mouse embryonic fibroblast (MEF) cells were maintained in DMEM media (Cat #11995, Gibco, CA, USA) containing 10% FBS (Thermo Fisher SCIENTIFIC, Waltham, MA, USA) and 0.1% gentamycin (Thermo Fisher SCIENTIFIC), and cells were passaged every three days. For the establishment of SIRT1-expressing cells, a pMFG-SIRT1 construct was used for producing lentivirus, and then lentivirus was infected into 293T cells and NIH3T3 cells. Puromycin was used for the selection of SIRT1 expression cells. To make inducible Ras cell lines, we replaced the attB gene in CSIV-TRE-RfA-UBC-KT vectors with the *RAS ^G12V^* gene, and then lentivirus was produced with these constructs. Lentivirus was infected into control NIH3T3 cells and SIRT1-expressing NIH3T3 cells, and then inducible-Ras cells were enriched by FACS sorting.

### 4.2. Reagents and Antibodies

Antibodies against phospho-ERK1/2 (T202/Y204) (#4370) and pan-acetyl lysin (#9441S) were obtained from Cell Signaling Technology (Danvers, MA, USA). Antibodies against β-actin (sc-47778), α-tubulin (sc-8035), ERK2 (sc-154), proliferating cell nuclear antigen (PCNA; sc-56), HA (sc-7392), Myc (sc-40), SIRT1 (sc-15404), HRAS (sc-520), and MKP1 (sc-370, sc-1199) were obtained from Santa Cruz biotechnology, Inc (Santa Cruz, CA, USA). Doxycyclin (Dox, D9891), Resveratrol (R5010), and (E/Z)-BCL hydrocholoride (B4313) were obtained from Sigma Aldrich (St. Louis, MO, USA), and U0126 (S1102) was purchased from Selleckchem (TX, USA). Nicotinamide (#481907) was purchased from Calbiochem (La Jolla, CA, USA).

### 4.3. Public Data Resources and Survival Analysis

RNA sequencing (RNA-seq) and clinical data of TCGA cohorts for 33 diverse cancer types were obtained by using the R package ‘TCGA biolinks’. A total of 9345 tumor patients with clinical information tracked over at least one month were used. Transcripts per million (TPM) and expected counts were taken as gene expression levels for survival analysis and differential gene expression analysis, respectively. Information on *KRAS* alteration status of each patient sample was obtained from cBioPortal (https://www.cbioportal.org/). As validation cohorts, lung cancer (*n* = 246) and breast cancer (*n* = 159) patients were used, and their *SIRT1* gene expression and clinical data were obtained from the Gene Expression Omnibus (GEO) database (GSE1456 and GSE31210, respectively).

For survival analysis, patients were divided into two groups according to the expression level of the gene of interest (e.g., *SIRT1* or *MKP1*) in each cohort. Using the median expression as a cutoff, the upper and lower groups were defined as ‘high’ and ‘low’ groups. Survival analysis was performed to test the difference in overall survival (OS) rate between the groups by using the R package ‘survival’. Hazard ratio (HR) and *p*-value (P) were computed using Cox proportional hazards regression analysis and log-rank test, respectively.

### 4.4. Gene Set Enrichment Analysis (GSEA)

Differential gene expression analysis between *SIRT1*-high and -low groups was performed using the R package ‘DESeq’, yielding a ranked list of genes. Using the gene list as input, the GSEAPreranked tool implemented in GSEA v4.0.2 software was run for the curated gene sets in the Molecular Signatures Database (https://www.gsea-msigdb.org/gsea/msigdb/). Significance was considered as nominal *p*-value < 0.05.

### 4.5. Transient Expression of Exogenous Protein

Transient expression was performed with Lipofectamin 2000 (Invitrogen, CA, USA) provided by manufacturer’s protocols. The DNA construct used is described as follows: Flag-SIRT1, G12V-RAS, N17-Ras, HA-MEK1 CA, HA-MEK1 DN, pCEP4-MKP1, pCEP4-MKP1 CS, and pCMV-p300. pCEP4-MKP1 and pCEP4-MKP1 CS vectors were kindly provided by professor Paul Shapiro (University of Maryland, School of Pharmacy, MD, USA).

### 4.6. Cell Proliferation, Clonogenic Assat, and Soft-Agar Assay

JuLI stage (NanoEntek, Waltham, MA, USA) was used to measure the cell proliferation rate. After cell seeding, plates were loaded on the JuLI stage for two days. With the JuLI stage, cell growth was monitored in timely dependent manner. For a clonogenic assay, 1 × 10^3^ cells were briefly cultured in six well plates, and 1 μg/mL doxycycline was treated in every two days. For soft-agar assay, 1 × 10^6^ cells were seeded in a 0.3% agar solution above 0.6% bottom agar. After a colony formed with both clonogenic and soft-agar assays, cells were rinsed with cold PBS and fixed with ice-cold methanol for 10 minutes. For visualization, colonies were stained with 0.1% crystal violet for 30 minutes. After this, plates with crystal violet were destained with PBS, and the numbers of colonies were measured with Image J software (National Institutes of Health, Bethesda, Maryland, USA, https://imagej.nih.gov/ij/).

### 4.7. RNA Preparation and Real-Time PCR

Total RNA was extracted by using easyBlue (cat #17061, iNtRON Biotechonology, Seongnam, Korea), followed by RT-PCR to generate the first strand cDNA (cat #RR036A, Takara, Shiga, Japan), and the cDNA was applied to real-time PCR with TB-Green (cat #RR420, Takara) under the manufacturer’s direction.

### 4.8. Immunoblotting and Immunoprecipitation

Cells were lysed with tissue lysis buffer supplemented with 0.2 mM sodium vanadate and 1 mM protease inhibitor cocktail (Roche, Basel, Switzerland), and for immunoblotting, an assay was performed in accordance with the standard method. For immunoprecipitation, 1 mg of total proteins were incubated with HA or Myc antibody at 4 °C overnight, followed by incubation with Protein G sepharose beads (GE healthcare Life Science, Sugar Notch, PA, USA) at 4 °C for additional four hours. The precipitates were washed with TLB for three times, following which immunoblotting was performed as described previously. The band intensity was measured using Image J software and normalized with the loading control. 

### 4.9. Statistical Analysis

The graphical data were presented as mean ± S.E.M. All the experiments were repeated at least three times and the representative results were presented. Statistical significance between groups was determined using Student’s t-test. Significance was assumed for *p* < 0.05 (*), *p* < 0.01 (**), *p* < 0.001 (***).

## 5. Conclusions

Taken together, these results suggest that cooperation of SIRT1 and MKP1 is crucial for suppressing the RAS-MEK-ERK axis-dependent tumorigenesis through modulating acetylation-phosphorylation crosstalk, which provides a novel strategy to manipulate RAS-dependent carcinogenesis. 

## Supplementary Materials

The following are available online at https://www.mdpi.com/2072-6694/12/4/909/s1, Figure S1: Prognostic significance of SIRT1 with KRAS mutation, Figure S2: Establishment of Ctrl-iRas and Sirt1-iRas cell lines, Figure S3: Regulation of phosphorylated ERK by the SIRT1 activity, Figure S4: SIRT1 directly binds to MKP1, Figure S5: Acetylation of MKP1 mediated by p300, Figure S6: MKP1 expression levels in various human cancer types, Figure S7: The whole western blot images of Figure 3A–F, Figure 4A–C, Figure 5A–C, Figure S2B, Figure S3A,B and Figure S4.
