# Supplementary material for: ERK Dephosphorylation through MKP1 Deacetylation by SIRT1 Attenuates RAS-Driven Tumorigenesis
Source: Cancers (Basel). 2020 Apr 8;12(4):909. doi: 10.3390/cancers12040909 (PMC7225992; doi:10.3390/cancers12040909)
Supplement: Supplementary file 1 [file cancers-12-00909-s001.pdf]

Supplementary Materials: ERK Dephosphorylation through MKP1 Deacetylation by SIRT1 Attenuates RAS-Driven Tumorigenesis

Ok-Seon Kwon, Haeseung Lee, Yun-Jeong Kim, Hyuk-Jin Cha, Na-Young Song and Mi-Ok Lee

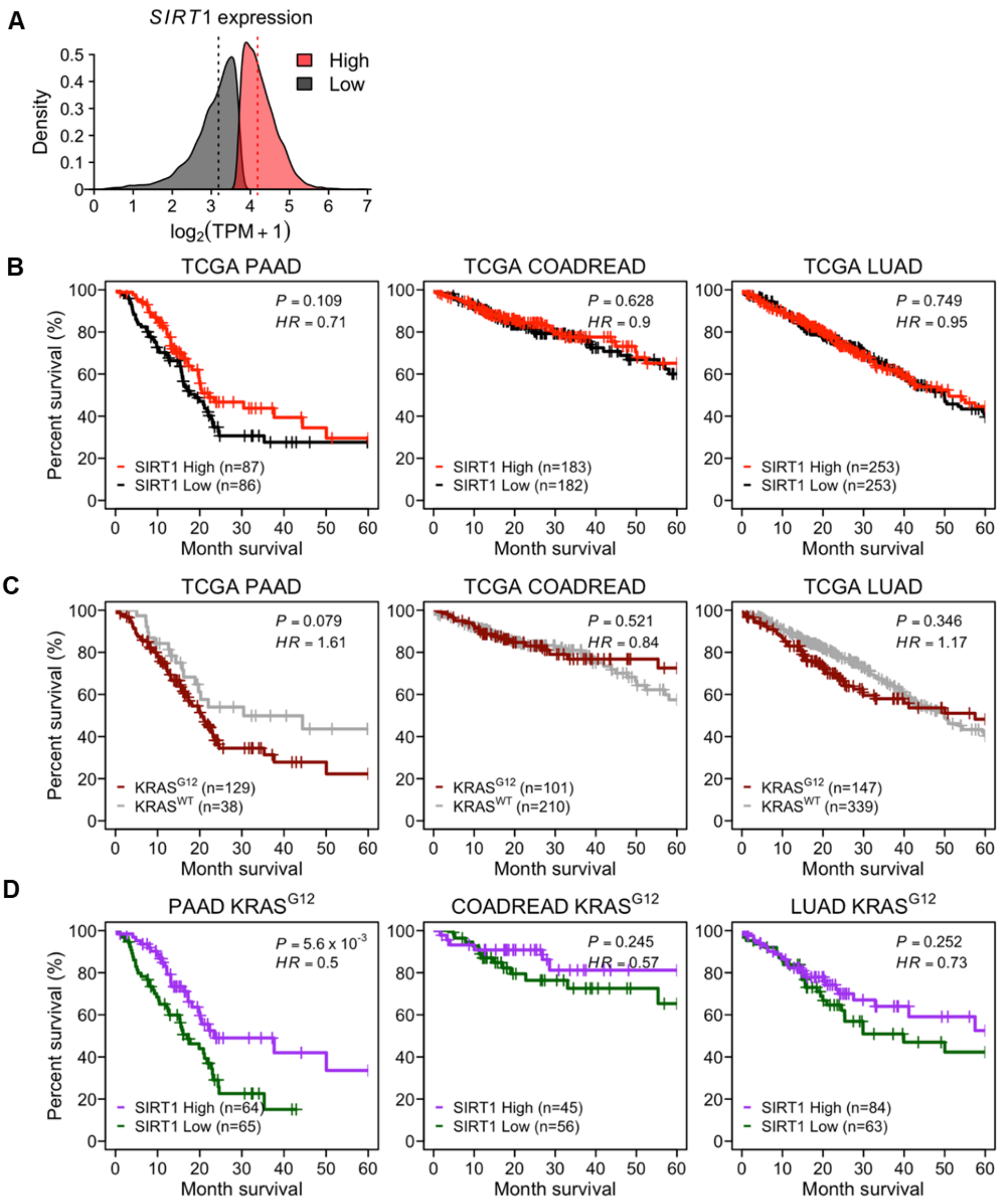

Figure S1. Protective role of *SIRT1* in cancer with *K-RAS* mutation. **(A)** Distribution of *SIRT1* gene expression levels of human cancers in TCGA Pan-cancer cohort. The RNA-seq data (log_2TPM) of 9,345 tumor samples comprising 33 cancer types are shown. SIRT1-high and -low groups were defined using the median expression as a cut-off. **(B)** The Kaplan-Meier curves showing the overall survival (OS) of cancer patients in the TCGA cohorts: pancreatic cancer (PAAD), colorectal cancer (COADREAD), and lung adenocarcinoma (LUAD). **(C)** The OS stratified by SIRT1-high and -low groups. **(D)** The OS stratified by *K-RAS* mutation status (wild-type or G12; the missense mutant that occurred at glycine 12). Hazard ratio (HR) and p-value (P) were calculated using Cox regression and log-rank test, respectively.

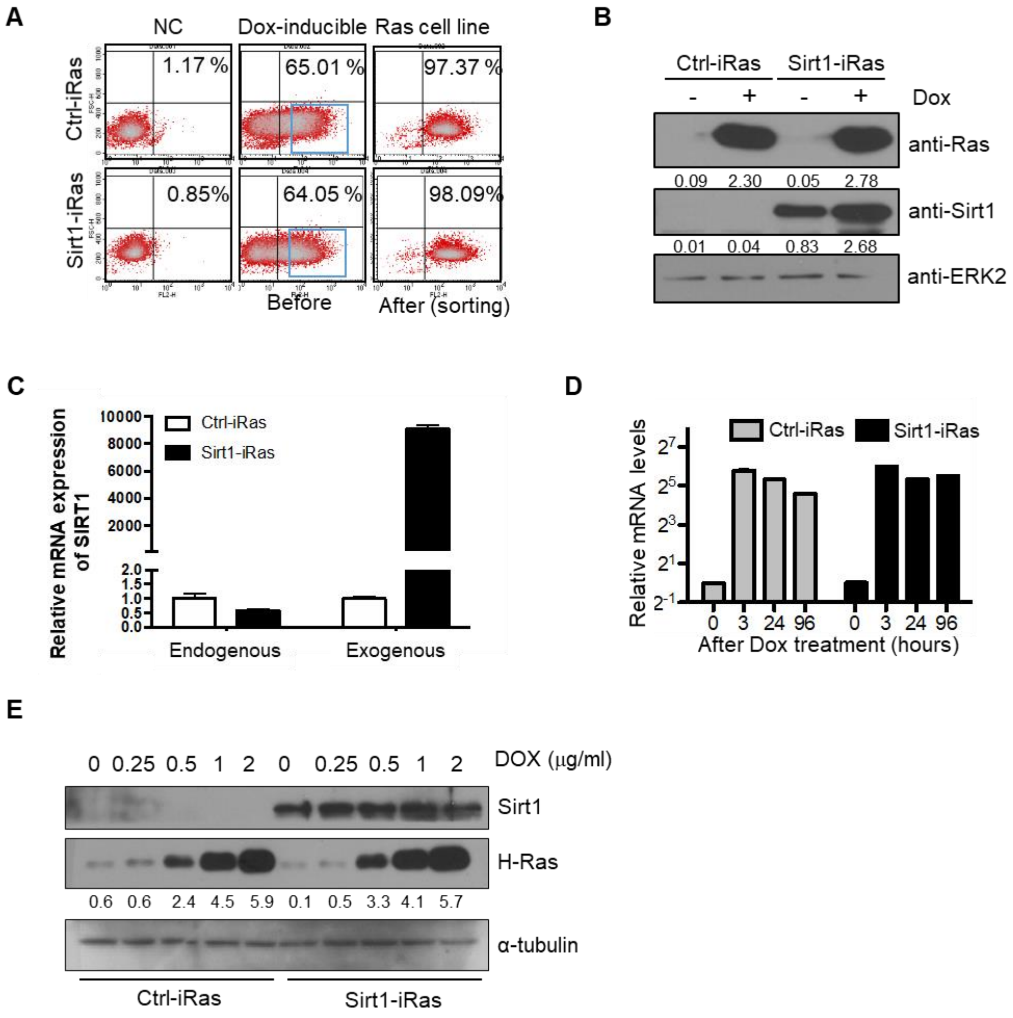

Figure S2. Establishment of Ctrl-iRas and Sirt1-iRas cell lines **(A)** Cell enrichment of the inducible H-Ras in control (Ctrl-iRas) or Sirt1 overexpression (Sirt1-iRas) NIH3T3 cells by flow cytometric cell sorting with humanized Kusabira-Orange fluorescence. **(B)** Immunoblotting (IB) analysis of H-Ras and Sirt1 expression in both Ctrl-iRas and Sirt1-iRas NIH3T3 cells after treatment with Dox. **(C)** The mRNA expression of endogenous (left) and exogenous (right) SIRT1 in Ctrl-iRas and Sirt1-iRas cells. **(D)** Relative mRNA levels of Ras were analyzed by qRT-PCR in Ctrl-iRas and Sirt1-iRas NIH3T3 cells at indicated time points following Dox treatment. **(E)** IB analysis for SIRT1 and H-RAS after Dox treatment (3 h) in a dose-dependent manner. α-tubulin, an equal loading control.

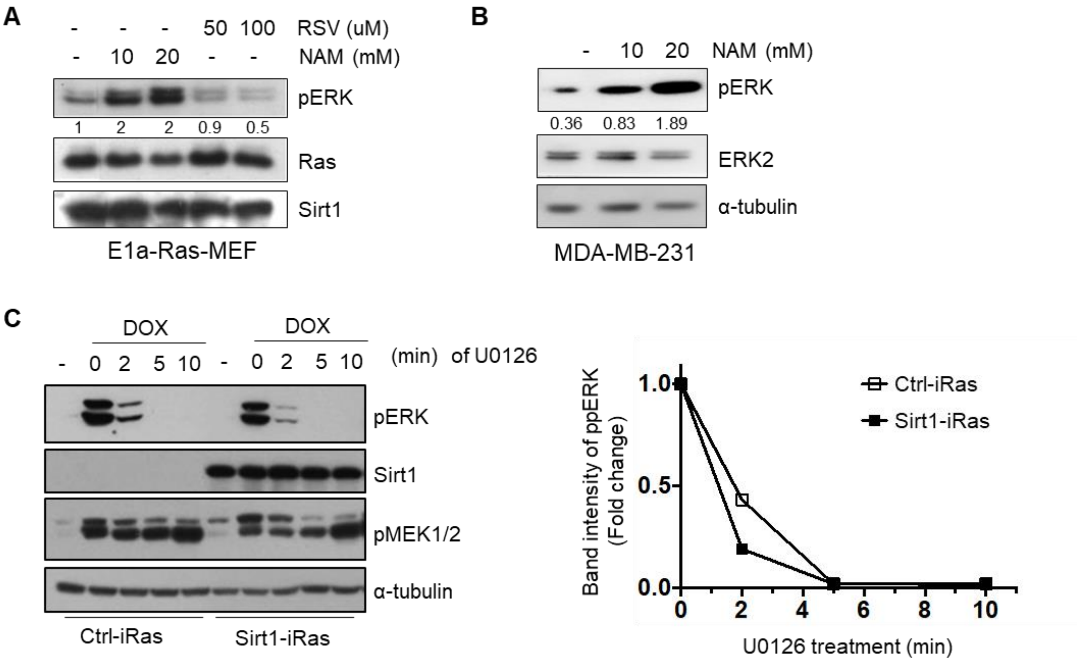

Figure S3. Regulation of phosphorylated ERK by the SIRT1 activity. (A) IB analysis of phospho-ERK, H-RAS, and SIRT1 in the E1a-Ras MEF cells after treatment with resveratrol (RSV) or nicotinamide (NAM). **(B)** IB analysis of phopho-ERK, ERK2 and α-tubulin in human breast cancer MDA-MB-231 cells after treatment with NAM for 24 h. **(C)** IB analysis of phopho-ERK, SIRT1, and phospho-MEK in Ctrl-iRas and Sirt1-iRas cells exposed to Dox (24 h) with or without U0126.

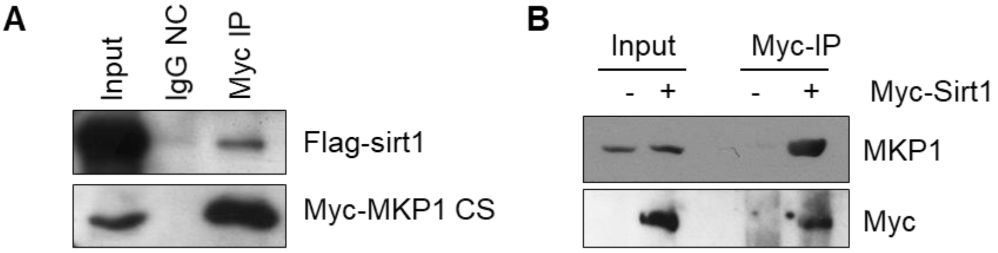

Figure S4. Sirt1 directly binds to MKP1. (**A)** The 293T cells were transiently transfected with Flag-tagged Sirt1 and Myc-tagged MKP1 CS and then IP was performed with an anti-Myc antibody, followed by IB with anti-Flag or anti-MKP1 antibodies. IgG, a negative control for IP. **(B)** The 293T cells were transiently transfected with the Myc-Sirt1 vector and then IP was performed with an anti-Myc antibody, followed by IB with an anti-MKP1 antibody.

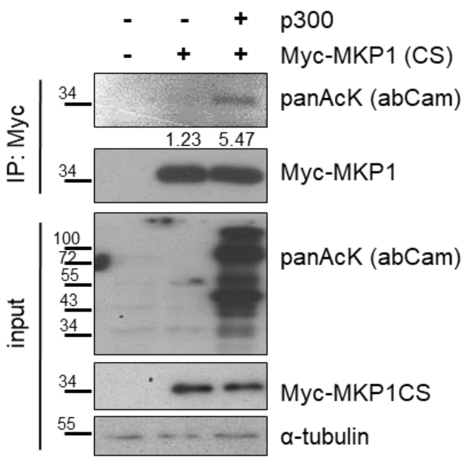

Figure S5. Acetylation of MKP1 mediated by p300. The 293T cells were transiently transfected with p300 and Myc-tagged MKP1 CS vectors and then IP was performed with an anti-Myc antibody, followed by IB with anti-Myc or anti-pan-acetyl lysine (panAcK) antibodies.

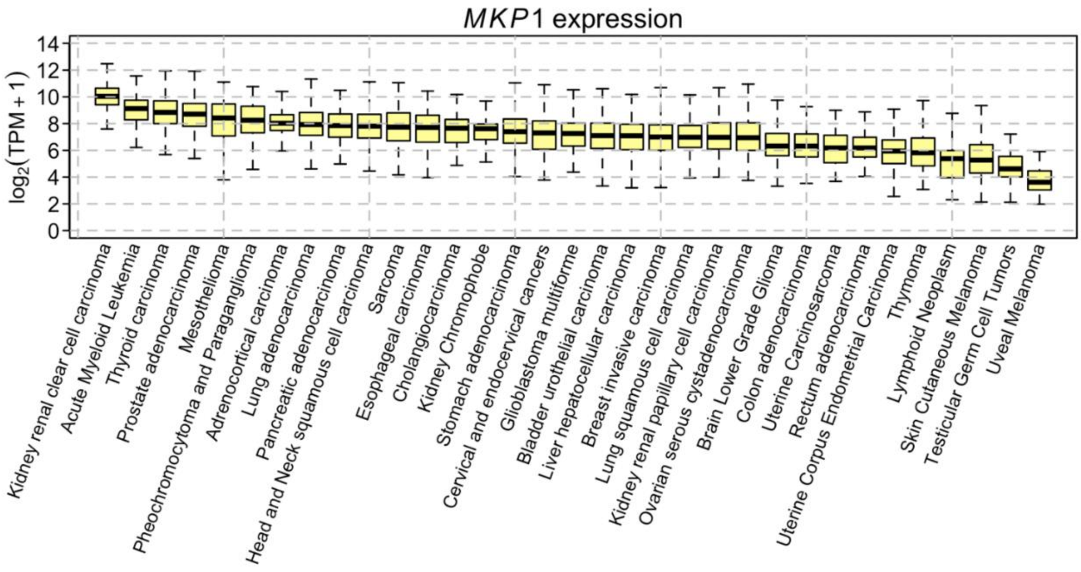

Figure S6. MKP1 expression levels in various human cancer types. Gene expression levels of *MKP1* in the TCGA pan-cancer cohort grouped by cancer type. The RNA-seq data (log_2TPM) of 9,345 tumor samples comprising 33 cancer types are shown. Cancer types were ordered by the median expression levels of MKP1.

HR and p-values calculated from each group comparison in Figure 6 are listed as follows:
SIRT1 high (MKP1 high vs low) = HR: 0.63, P: 0.09
SIRT1 low (MKP1 high vs low) = HR: 0.83, P: 0.37
MKP1 high (SIRT1 high vs low) = HR: 0.47, P: 0.0024
MKP1 low (SIRT1 high vs low) = HR: 0.61, P: 0.034
SIRT1 high, MKP high vs SIRT1 low, MKP low = HR: 0.39, P: 0.000019.

Figure 3A

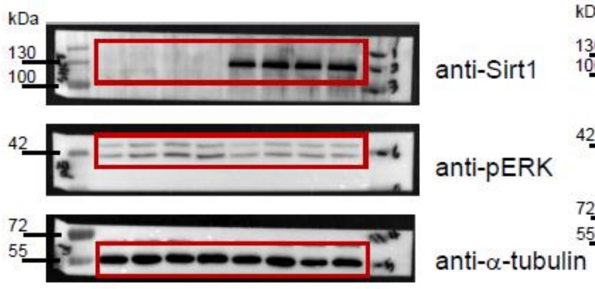

Figure 3B

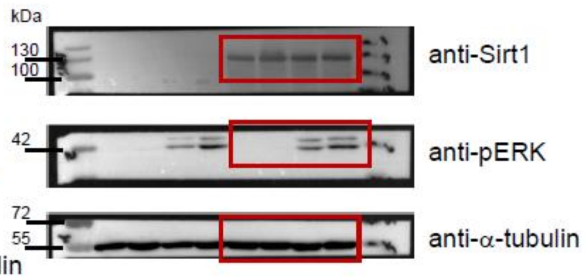

Figure 3C

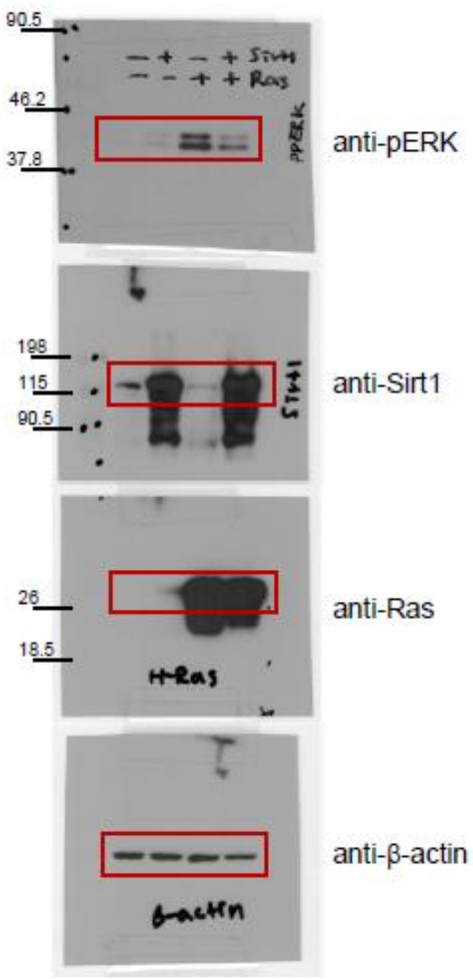

Figure 3D

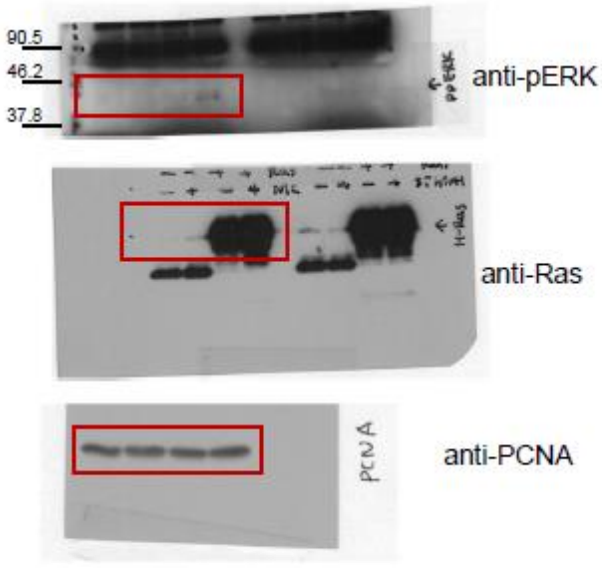

Figure 3E

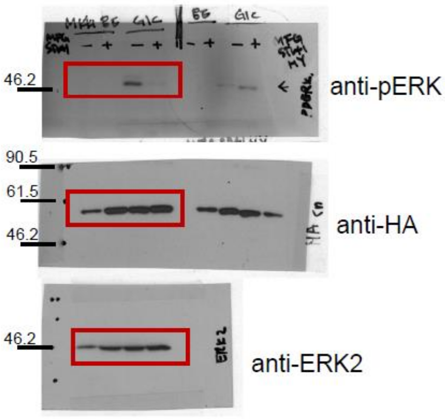

Figure 3F

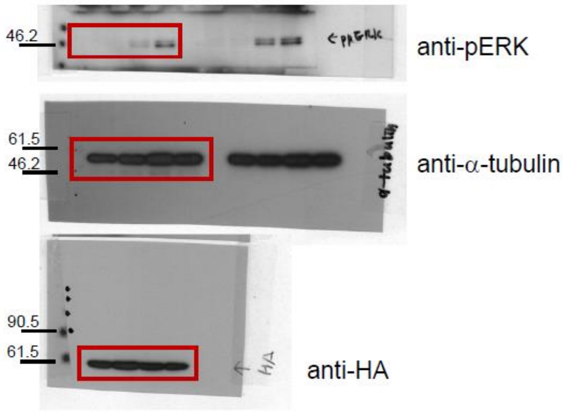

Figure 4A

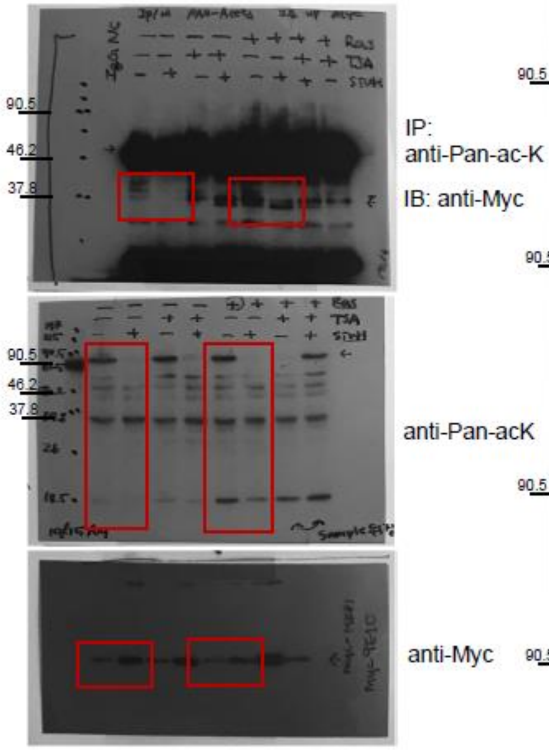

Figure 4B

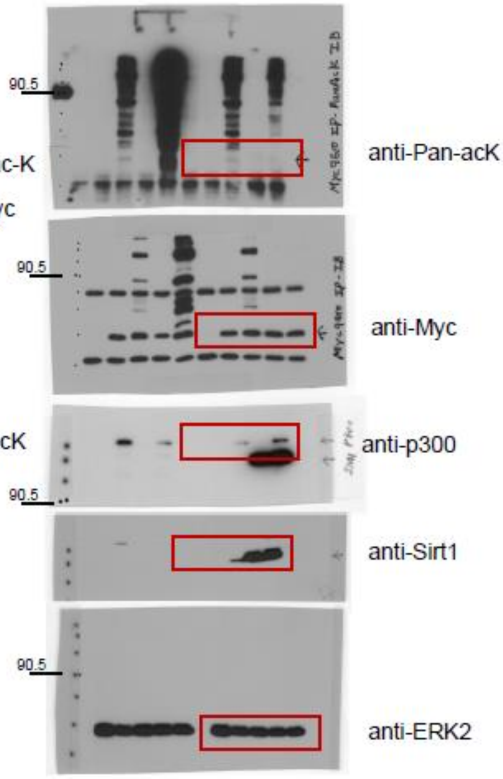

Figure 4C

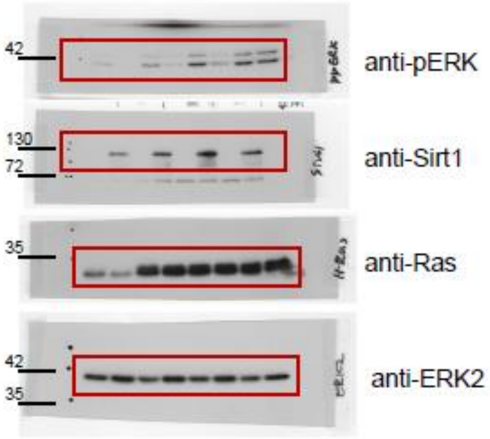

Figure 5A

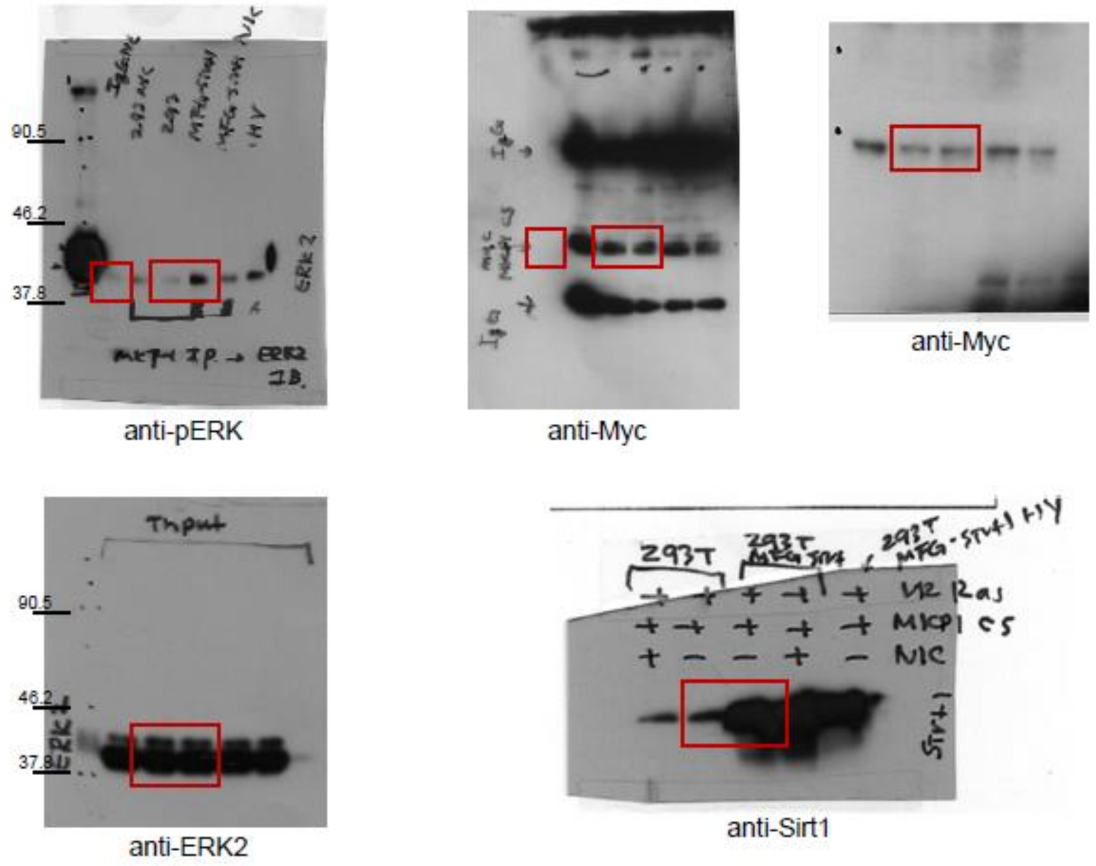

Figure 5B

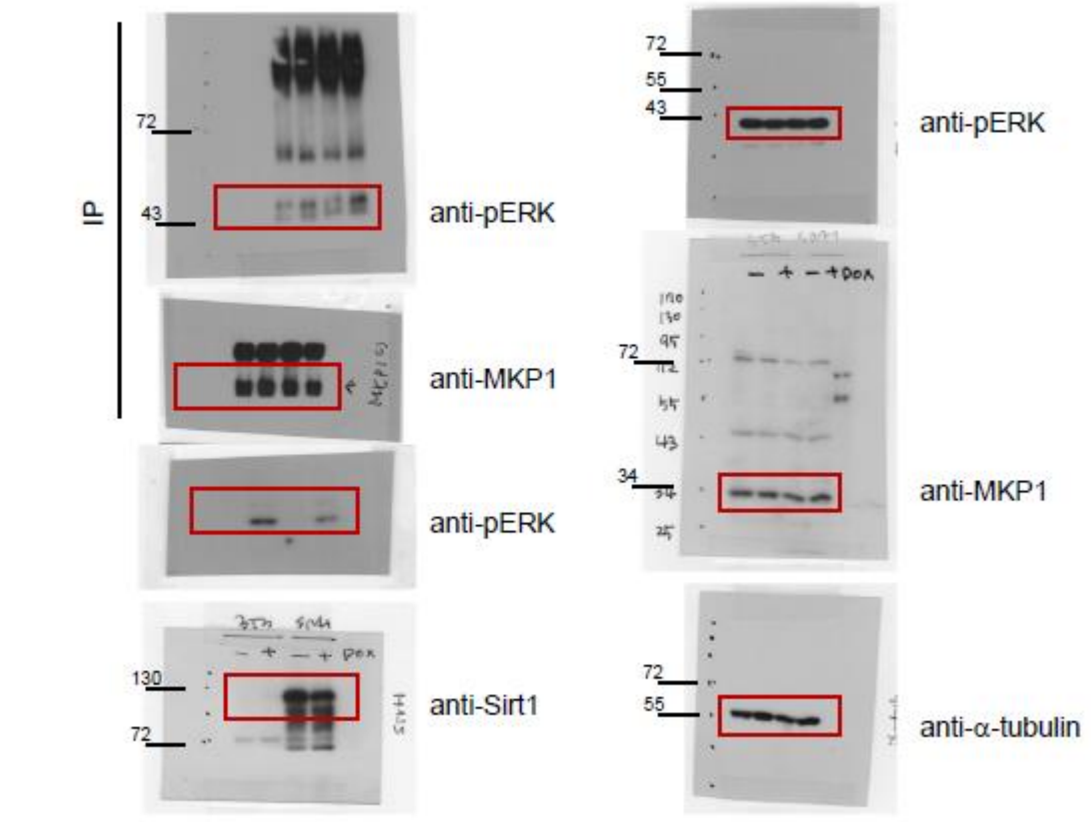

Figure 5C

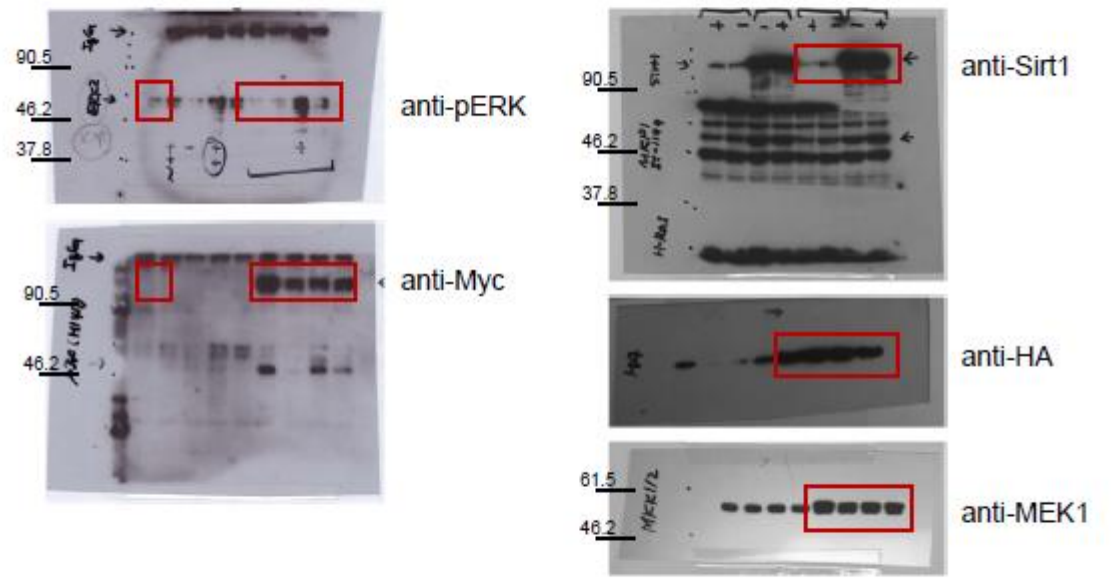

Figure S2B

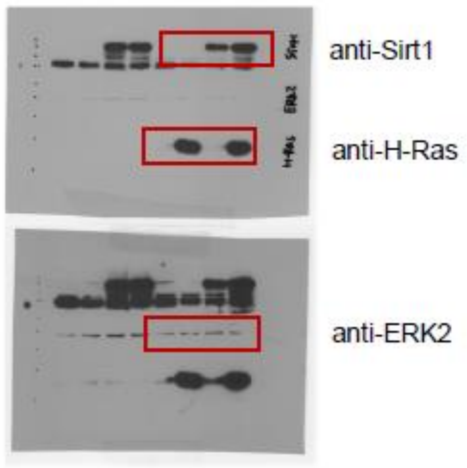

Figure S3A

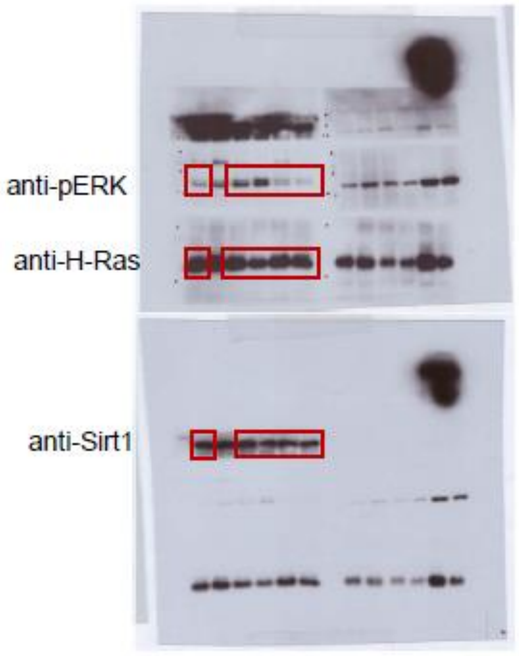

Figure S3B

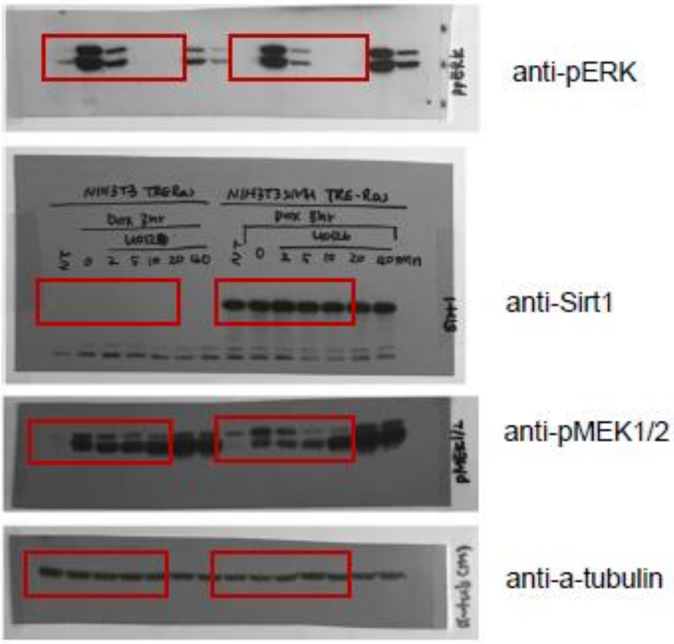

Figure S4

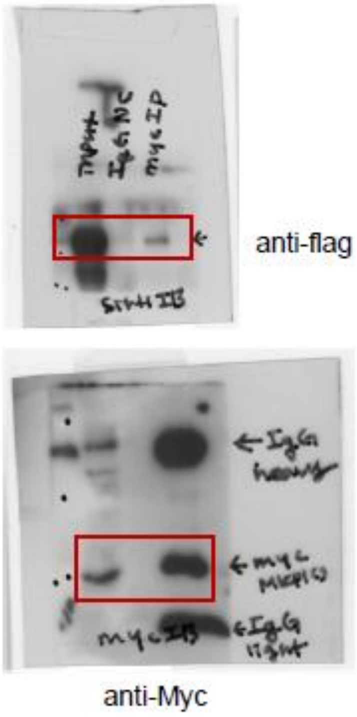

Figure S7. The whole western blot images of Figure 3A–F, Figure 4A–C, Figure 5A–C, Figure S2B, Figure S3A,B and Figure S4.

© 2020 by the authors. Licensee MDPI, Basel, Switzerland. This article is an open access article distributed under the terms and conditions of the Creative Commons Attribution (CC BY) license (http://creativecommons.org/licenses/by/4.0/).
